# Millimeter Wave High Resolution Radar Accuracy in Fog Conditions—Theory and Experimental Verification

**DOI:** 10.3390/s18072148

**Published:** 2018-07-04

**Authors:** Yosef Golovachev, Ariel Etinger, Gad A. Pinhasi, Yosef Pinhasi

**Affiliations:** Faculty of Engineering, Ariel University, Ariel 40700, Israel; yosi_habad@yahoo.com (Y.G.); etinger7@ariel.ac.il (A.E.); gadip@ariel.ac.il (G.A.P.)

**Keywords:** extremely high frequencies, FMCW radar, atmosphere, millimeter waves, Tera-Hertz frequencies

## Abstract

Attenuation and group delay effects on millimeter wave (MMW) propagation in clouds and fog are studied theoretically and verified experimentally using high resolution radar in an indoor space filled with artificial fog. In the theoretical analysis, the frequency-dependent attenuation and group delay were derived via the permittivity of the medium. The results are applied to modify the millimeter-wave propagation model (MPM) and employed to study the effect of fog and cloud on the accuracy of the Frequency-Modulated Continuous-Wave (FMCW) radar operating in millimeter wavelengths. Artificial fog was generated in the experimental study to demonstrate ultra-low visibility in a confined space. The resulted attenuation and group delay were measured using FMCW radar operating at 320–330 GHz. It was found that apart from the attenuation, the incremental group delay caused by the fog also played a role in the accuracy of the radar. The results were compared to the analytical model. It was shown that although the artificial fog has slight different characteristics compare to the natural fog and clouds, in particle composition, size, and density, the model predictions were good, pointing out that the dispersive effects should be considered in the design of remote sensing radars operating in millimeter and sub-millimeter wavelengths.

## 1. Introduction

The extremely high frequencies above 30 GHz, known as millimeter waves, cover a wide range of the electromagnetic spectrum. Many applications, such as mobile wireless communications [1,2], satellite communications [3] mobile communication, road traffic safety radars [4,5], and remote (imaging and non-imaging) sensing, as well as wireless power transfer (WPT), are all being considered to use this relatively free frequency regime. Wide frequency bands in millimeter waves allows a high bit rate in digital communication links and high distance resolution in radar systems to be obtained [6].

However, when millimeter-wave radiation propagates through the atmosphere, it suffers from molecular absorption and refraction [7,8,9]. The gas composition and meteorological conditions of the atmosphere have frequency-dependent effects on the millimeter wave propagation [10,11,12]. In particular, the presence of suspended water droplets, like in fog and clouds, may be one of the major factors for attenuation and dispersion effects on millimeter wave signals [13,14,15]. In radar systems that operate in millimeter and sub-millimeter wavelengths, atmospheric dispersion plays an important role in the accuracy of distance measurements, as discussed in [16].

The atmospheric frequency response is being studied intensively both theoretically and experimentally. However, vast majority of the works refer to the attenuation effect only. The signal phase shift and time delay effects are usually ignored, although they can be significant in extreme conditions. Furthermore, works on the propagation in dielectric media sometimes using equivalent but different terms to describe the problem, such as medium permittivity, refractivity, and susceptibility.

In the present work, the effect of suspended droplets was studied theoretically and experimentally to demonstrate the effect of extremely low visibility conditions on the signal strength and time delay. First, the propagation factors are presented as analytic expressions of the permittivity and refractivity, and the relation between them. Second, a modified millimeter-wave propagation model (MPM) is employed for the prediction of the suspended water droplets effect. Finally, an experimental verification of the effect on an MMW radar signal strength and time delay is presented. We used a dense artificial fog, created by a fog machine, to demonstrate the effects quantitatively. The results are compared to the analytical model predictions. In order to set the definitions employed along the paper, we start from a short review of field propagation in dielectrics in general and then focus on the atmospheric medium.

## 2. Propagation in Dielectric Media

An electromagnetic wave propagating in a dielectric medium such as the atmosphere is being affected by losses and time delay. These effects are described by the propagation expression for the electric field in the frequency domain. Propagating a distance d in a homogeneous, linear medium, the resulted field is:

(1) $${\tilde{E}}_{out}\left(f\right)={\tilde{E}}_{in}\left(f\right)e^{-jk\left(f\right)\cdot d}$$

where ${\tilde{E}}_{in}\left(f\right)$ and ${\tilde{E}}_{out}\left(f\right)$ are the transmitted and received fields respectively, presented as phasors in the frequency domain and:

(2) k(f)=−jα(f)+β(f)

is a frequency-dependent propagation factor, composed of the attenuation per unit length α(f)=−Im{k(f)} and the wavenumber β(f)=Re{k(f)}. The group delay at a distance d can be found from the derivative of the wavenumber:

(3) $$\tau_{d}\left(f\right)=\frac{d}{2\pi }\frac{d\beta }{df}=\frac{d}{c}+\Delta \tau_{d}\left(f\right)$$

where $c\approx 2.997\times 10^{8}\;m/s$ is the speed of light in a vacuum. The group delay can be described as a summation of the constant delay $\tau_{0}=d/c$ in vacuum and a frequency-dependent incremental part $\Delta \tau_{d}\left(f\right)$.

In the first stage of the model description, the properties of the atmosphere as a dielectric medium are presented in terms of its permittivity. In the second stage, the permittivity of a suspended droplets atmospheric medium as in foggy or cloudy conditions is discussed. Finally, we derive the parameters of millimeter wave propagation in such conditions.

The propagation factor is commonly expressed via the dielectric medium properties, either relative permittivity $\varepsilon_{r}\left(f\right)$ or index of refraction n(f):

(4) $$k\left(f\right)=\frac{2\pi f}{c}\underset{n\left(f\right)}{\underset{︸}{\sqrt{\varepsilon_{r}\left(f\right)}}}$$

where *c* ≈ 2.997 × 10^8^ m/s is the speed of light in a vacuum. The relative permittivity of the medium is a complex, frequency dependent quantity:

(5) $$\varepsilon_{r}\left(f\right)=\varepsilon^{\prime }\left(f\right)-j\varepsilon^{\prime \prime }\left(f\right)$$

with real $\varepsilon^{\prime }\left(f\right)$ and imaginary $\varepsilon^{\prime \prime }\left(f\right)$ parts. The attenuation α(f) and the wavenumber β(f) are related to the permittivity via two coupled equations:

(6) $$\begin{array}{l} \alpha^{2}\left(f\right)-\beta^{2}\left(f\right)=-{\left(\frac{2\pi \;f}{c}\right)}^{2}\varepsilon^{\prime }\left(f\right)\\ 2\alpha \left(f\right)\;\beta \left(f\right)={\left(\frac{2\pi \;f}{c}\right)}^{2}\varepsilon^{\prime \prime }\left(f\right) \end{array}$$

The last equations have two pairs of solutions:

(7) $$\alpha_{1,2,3,4}=\pm \frac{1}{\sqrt{2}}\left(\frac{2\pi \;f}{c}\right)\sqrt{-\varepsilon^{\prime }\pm \sqrt{{\left(\varepsilon^{\prime }\right)}^{2}+{\left(\varepsilon^{\prime \prime }\right)}^{2}}}\;\beta_{1,2,3,4}=\pm \frac{1}{\sqrt{2}}\left(\frac{2\pi \;f}{c}\right)\varepsilon^{\prime \prime }\frac{1}{\sqrt{-\varepsilon^{\prime }\pm \sqrt{{\left(\varepsilon^{\prime }\right)}^{2}+{\left(\varepsilon^{\prime \prime }\right)}^{2}}}}$$

Since the attenuation α(f) and the wavenumber β(f) are real and positive numbers, the physical solution should be [17]:

(8) α(f)=−Im{k(f)}=2π fcε′(f)2[1+(ε″(f)ε′(f))2−1]β(f)=Re{k(f)}=2π fcε′(f)2[1+(ε″(f)ε′(f))2+1]

Using the last Expression (8), the frequency dependent incremental part of the time delay (3) can be derived analytically:

(9) $$\Delta \tau_{d}\left(f\right)=\frac{d}{2\pi }{\left(\frac{2\pi \;f}{c}\right)}^{2}\frac{1}{\alpha^{2}+\beta^{2}}\left[\alpha \left(\frac{\varepsilon^{\prime \prime }}{f}+\frac{1}{2}\frac{d\varepsilon^{\prime \prime }}{df}\right)+\beta \left(\frac{\varepsilon^{\prime }}{f}+\frac{1}{2}\frac{d\varepsilon^{\prime }}{df}\right)\right]-\frac{d}{c}$$

The above analytical Expressions (8) and (9) describe the three major physical phenomena of the electromagnetic field propagation in dielectric media: attenuation, phase dispersion, and the resulted group delay.

Propagation of light as well as millimeter and sub-millimeter wave infra-red radiation in the atmosphere are usually studied using the complex refractive index n(f), which is written in terms of the refractivity, N(f) (given in ppm):

(10) $$n\left(f\right)=1+N\left(f\right)\times 10^{-6}$$

The frequency dependent refractivity is complex and can be presented as:

(11) $$N\left(f\right)=N_{0}+N^{\prime }\left(f\right)-jN^{\prime \prime }\left(f\right)$$

where the nondispersive part *N*_0_ is real and positive and the other two terms, the real *N*’(*f*) and the imaginary *N*”(*f*) parts, are frequency dependent. The relations between the complex refractivity and complex permittivity can be obtained by using Equations (4), (5), (10) and (11):

(12) $$n\left(f\right)=\sqrt{\varepsilon^{\prime }\left(f\right)-j\varepsilon^{\prime \prime }\left(f\right)}=1+[N_{0}+N^{\prime }\left(f\right)-jN^{\prime \prime }\left(f\right)]\times 10^{-6}$$

resulting in an expression for the real part:

(13) $$\varepsilon^{\prime }\left(f\right)=1+2[N_{0}+N^{\prime }\left(f\right)]\times 10^{-6}$$

and for the imaginary part:

(14) $$\varepsilon^{\prime \prime }\left(f\right)=2N^{\prime \prime }\left(f\right)\times 10^{-6}$$

Since the dispersion models often use combinations of these quantities, permittivity, and refractivity, the above relations enable one to fuse between the models and present a generalized one. The media properties can be also presented in terms of the refractivity:

(15) α(f)=−Im{k(f)}=2π fcN″(f)×10−6β(f)=Re{k(f)}=2π fc[(1+N0×10−6)+N′(f)×10−6]τd(f)=d2πdβdf=dc{(1+N0×10−6)+[N′(f)+fdN′df]×10−6}

For the study of millimeter waves propagation in the atmosphere, the MPM is employed [8,9]. In these models, quantitative values of the dispersive complex refractivity (11) are given via the permittivity of the gases and water droplets composing the atmosphere. The refractivity is represented as a summation of five terms:

(16) $$N(f)=\left(N_{L}+N_{d}+N_{c}\right)+N_{W}+N_{R}$$

**Figure 1:**
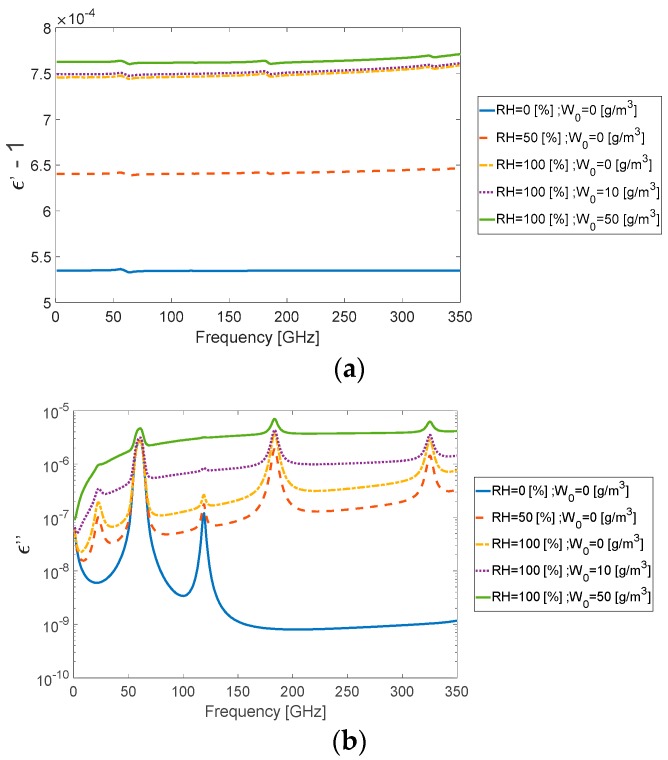
(**a**) The real $\varepsilon^{\prime }\left(f\right)$ and (**b**) imaginary $\varepsilon^{\prime \prime }\left(f\right)$ part of dielectric permittivity for different relative humidity *RH* (%) and water droplet concentration $W_{0}$ (g/m^3^) values.

Here $N_{L}\left(f\right)$ is moist air resonance contributions, $N_{d}\left(f\right)$ is dry air non-resonant spectra, $N_{c}\left(f\right)$ is water vapor continuum spectrum, $N_{W}\left(f\right)$ is suspended water-droplet refractivity and $N_{R}\left(f\right)$ is rain approximation. It is important to note that the different refractivity terms above are mutually independent and according to Equation (16) they are accumulated additively, contributing to the overall refractivity. A comparative study between the comprehensive MPM and the International Telecommunication Union (ITU) recommendation reveals that although the absorption peaks of water vapor and oxygen are not taken into account in the ITU model, there is good fit between the models [18]. From evaluation of the refractivity terms (16), it was found that the suspended water droplets had a major effect on the attenuation while the significant effect on the group delay was due to air humidity $N_{L}\left(f\right)$ [19]. The dielectric permittivity for different values of relative humidity RH and water droplet concentration $W_{0}$ is presented in Figure 1, for real $\varepsilon^{\prime }\left(f\right)$ and imaginary $\varepsilon^{\prime \prime }\left(f\right)$ parts. Note that Figure 1b describes the imaginary part $\varepsilon^{\prime \prime }\left(f\right)$ of the atmospheric permittivity is in a logarithmic scale. It seems that the fog effects increased intensely with frequency.

**Figure 2:**
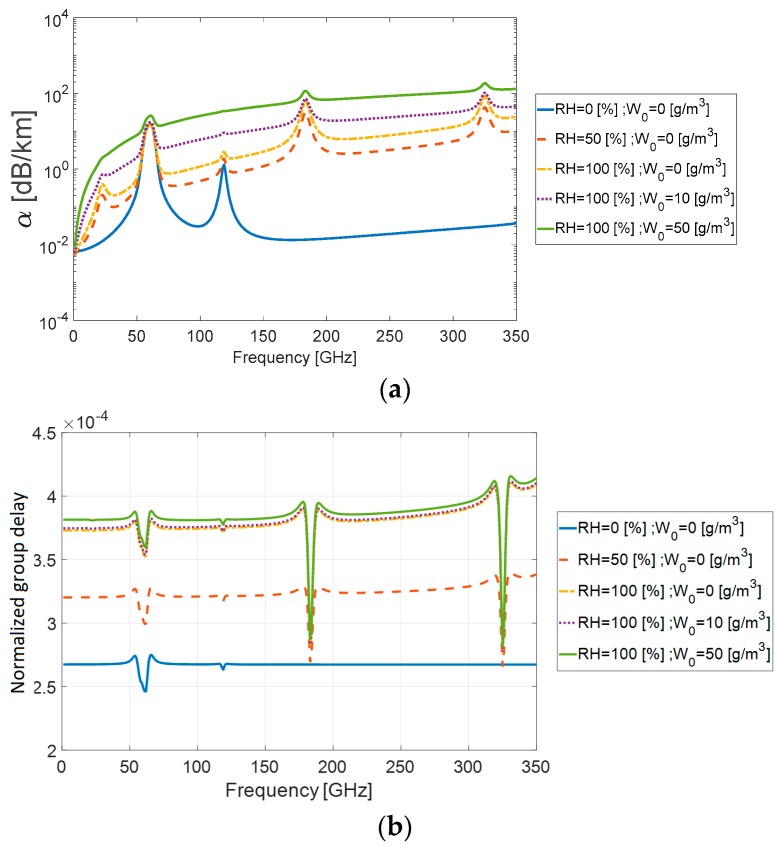
(**a**) Attenuation coefficient 20log(e)⋅α(f) in (dB/km) and (**b**) normalized group delay $c\cdot \Delta \tau_{d}\left(f\right)/d-1$ for different relative humidity *RH* (%) and water droplet concentration $W_{0}$ (g/m^3^) values.

Substituting $\varepsilon^{\prime }\left(f\right)$ and $\varepsilon^{\prime \prime }\left(f\right)$ into Equations (8) and (9), we calculated the attenuation coefficient α(f), the wavenumber β(f), and the resulted frequency dependent incremental part of group delay $\Delta \tau_{d}\left(f\right)$. The attenuation 20log(e)⋅α(f) in (dB/km) and the dimensionless measure $c\cdot \Delta \tau_{d}\left(f\right)/d-1$ for different values of relative humidity RH and water droplet concentration $W_{0}$ are presented in Figure 2.

## 3. Fog Characterization

Fog and clouds contain water droplets or ice crystals suspended in the air. They are normally formed at a relative humidity *RH* near 100%. The fog often is characterized via parameters like visibility, Vis (measured in (m)), droplet number concentration $n_{d}$ (measured in (cm^−3^)), and mass liquid water content $W_{0}$ (measured in (g/m^3^)). The parametrization of the suspended water droplets (SWD) is required for the theoretical and experimental study.

The visibility in the current work is defined as the distance at which visible light is attenuated to 2% of the maximum light intensity attained in clear sky. According to the International Commission on Illumination, this definition presents the visual range where the contrast ratio for a black target of a “reasonable” size against the horizon viewed by a typical human eye falls down to 0.05 [20].

**Figure 3:**
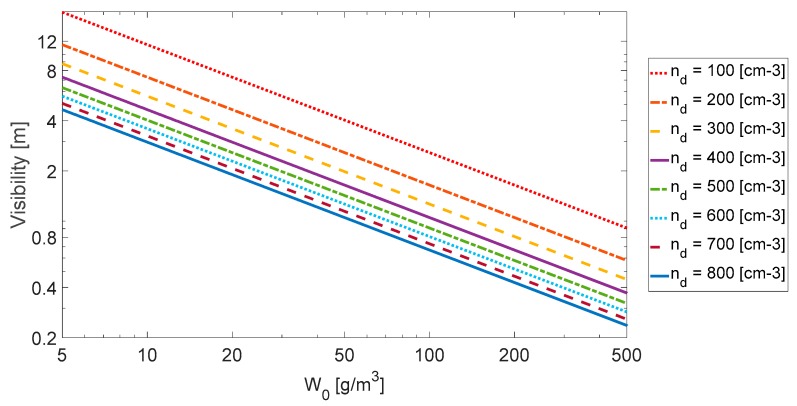
Visibility (m) as a function of liquid water content $W_{0}$ (g/m^3^) on assumed droplet number concentration $n_{d}$ (cm^−3^).

The suspended water droplets size in typical fog and clouds is in the range of 5–50 μm and the number concentration of droplets is in the range 10^2^–10^3^ cm^−3^ [11,21,22,23]. In heavy fog the numbers may increase even further. The mass liquid water content is typically 0.05 g/m^3^ for medium fog (visibility of the order of 300 m) and 0.5 g/m^3^ for heavy fog (visibility of the order of 50 m). Gultepe et al. [22] developed a generalized expression connecting between visibility, droplet number concentration $n_{d}$ and liquid water content $W_{0}$:

(17) $$Vis=1002\cdot {\left(n_{d}\cdot W_{0}\right)}^{-0.6473}$$

where the visibility is given in (km). The product ${\left(n_{d}\cdot W_{0}\right)}^{-1}$ is the termed “fog index”. This model can roughly estimates the visibility with more than 50% uncertainty depending on environmental conditions [23]. Graphs of the visibility as a function of liquid water content *W_0_* are drawn in Figure 3 for different number concentrations $n_{d}$.

Table 1 summarizes some of the measurements carried out experimentally in previous studies. Millimeter wave attenuations measured in different monitored foggy conditions, are presented. In these works, quantitative measurements of the liquid water content $W_{0}$ were also performed, enabling us to demonstrate a comparison with the fog characterization model.

## 4. The Effect of Fog on Frequency-Modulated Continuous-Wave (FMCW) Radar Accuracy

Previous theoretical work indicated that dispersion in the atmospheric medium affects the accuracy of radars [19]. In the setup described in the followings, a wide band FMCW is utilized for an experimental study of the effect of fog on the accuracy of distance measurement to a target. The transmitted signal is a frequency modulated (FM) signal with an instantaneous frequency varying linearly in time (chirp) [19]. The time dependent frequency in the chirp is given as:

(18) $$f_{i}\left(t\right)=f_{0}+\frac{\mathrm{BW}}{T_{s}}\cdot t$$

where $f_{0}$ is the start frequency of the chirp, BW is frequency span, and $T_{s}$ is sweep time. Passing through the atmosphere to the target located at a distance d, scattered and reflected back to the radar receiver, the ‘chirped’ signal is delayed and its intensity is reduced. The intermediate frequency (IF) of the detected signal obtained at the receiver output is expected to be:

(19) $$f_{IF}=2\cdot \frac{\mathrm{BW}}{T_{s}}\cdot \tau_{d}=2\cdot \frac{\mathrm{BW}}{T_{s}}\left(\frac{d}{c}+\Delta \tau_{d}\right)$$

Here $\tau_{d}$ is the time delay in propagation a range d  to the target. For propagation in a vacuum, the incremental group delay $\Delta \tau_{d}=0$, and the resulted IF signal would have been a single frequency tone at:

(20) $$f_{m}=2\cdot \frac{\mathrm{BW}}{T_{s}}\cdot \frac{d}{c}$$

which is proportional to the target distance *d*. However, when a dispersive medium (as in foggy conditions) is involved, there is a shift in the intermediate frequency due to the incremental group delay $\Delta \tau_{d}$:

(21) $$\Delta f_{IF}=f_{IF}-f_{m}=2\cdot \frac{\mathrm{BW}}{T_{s}}\cdot \Delta \tau_{d}$$

Using the Expression (9) we derived for group delay $\Delta \tau_{d}$, one can express the error Δd in the radar range measurement expected due to the dielectric properties of the medium in different weather conditions:

(22) $$\frac{\Delta d}{d}=c\cdot \frac{\Delta \tau_{d}\left(f_{0}\right)}{d}=2\pi \left(\frac{f_{0}{}^{2}}{c}\right)\frac{1}{\alpha^{2}+\beta^{2}}\left[\alpha \left(\frac{\varepsilon^{\prime \prime }}{f_{0}}+\frac{1}{2}{\frac{d\varepsilon^{\prime \prime }}{df}|}_{f_{0}}\right)+\beta \left(\frac{\varepsilon^{\prime }}{f_{0}}+\frac{1}{2}{\frac{d\varepsilon^{\prime }}{df}|}_{f_{0}}\right)\right]-1$$

## 5. Experimental Setup

Now we demonstrate the effect described in the preceding sections using a high resolution FMCW radar operating at the higher band of the millimeter waves, at 330 GHz [27]. A set of experiments have been conducted for studying the effect of fog on the radar performances even at very low visibility conditions, down to 0.5 m. The results were used to demonstrate the effects and validate the model for such extreme conditions. The experimental setup was based on a radar system placed in a confined indoor space filled with artificial fog. The radar system and a metal target were placed in the two far ends of the closed corridor. The fog was created by a thermal fog machine. The chamber has a dimension of 20 m × 3.5 m × 4 m, where the distance between the radar and the target was *d* = 18.8 m.

**Figure 4:**
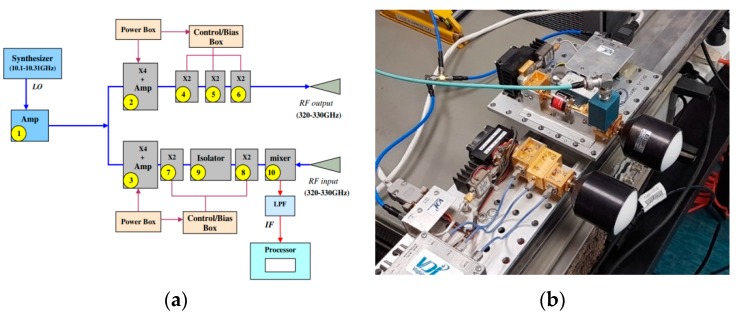
The 320–330 GHz Frequency-Modulated Continuous-Wave (FMCW) radar used in the experimental setup: (**a**) Block diagram and (**b**) Photo including transmitting and receiving antennas.

Schematics for the FMCW radar used in the experiment is given in Figure 4a. The HP-8350B frequency-sweeping synthesizer is employed as a primary driver of the Local Oscillator (LO). Its frequency was tuned to sweep from 10 to 10.31 GHz and multiplied by a factor 32 providing linear FM signal starting from $f_{0}=320\;\mathrm{GHz}$ with a sweep of BW=10 GHz. The transmission power was 10 dBm. Both the transmitting and receiving antennas were ELVA-1 custom design Gaussian horn-lens antenna with a gain of 40 dBi and linear horizontal polarization. A photograph of the 320–330 GHz FMCW radar is given in Figure 4b. The operational parameters of radar are summarized in Table 2. The detected signal obtained at the output of the harmonic mixer at the receiver chain was analyzed using a spectrum analyzer, model R&S FSV40. The spectrum of the detected signal was measured to find strength of the IF signal and its spectral components.

A convenient way to create stable, sustainable, homogeneous, low visibility fog in a relatively large indoor space is by using artificial fog. The artificial fog was generated using a fog machine type MAGNUM 850 with maximum fog output of 200 m^3^ per minute. The water based aerosol fog was created by using vaporizing proprietary water and glycol-based (glycols, poly-glycols) fog juice [28]. According to the manufacturer, the particle size produced by this thermal fog machine, was within the wide range of 0.25–60 µm [29].

**Figure 5:**
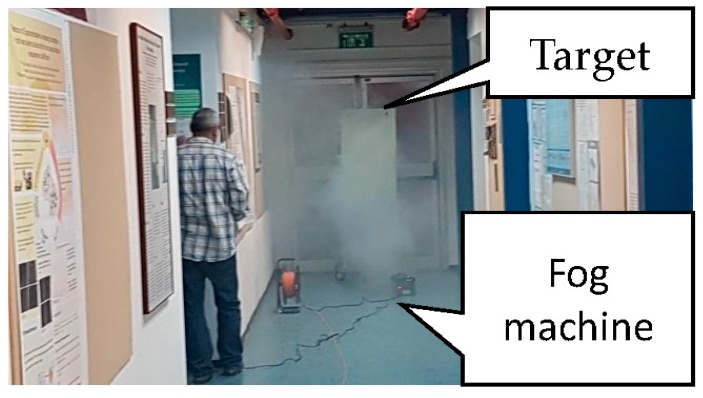
The corridor and the fog streaming start-up.

Although the artificial fog used in the current experiment was somewhat different in its characteristics from ‘natural’ water fog and clouds, its small particle sizes fit to our experimental requirements. The typical droplet size in natural fog is about 10 µm. The artificial fog droplet diameter was measured to be around $R_{p}=4\;\mu m$ [28]. This leads to a size parameter $2\pi R_{p}/\lambda =0.1$ at 330 GHz. This is well below the Mie scattering regime and within the Rayleigh approximation. The spatial fog spread in space was homogenized using a series of small fans along the corridor (see Figure 5).

The composition of the artificial fog was based on aqueous glycol solution. The solution components and their respective refractive index were triethylene glycol (*n* = 1.4531), 1,3-butylene glycol (1.4401), propylene glycol (1.4324), and deionized water (1.330). Since the weight percentage of each component was not provided by the manufacturer, an estimation was made for the refractive index as 1.439 [29].

**Figure 6:**
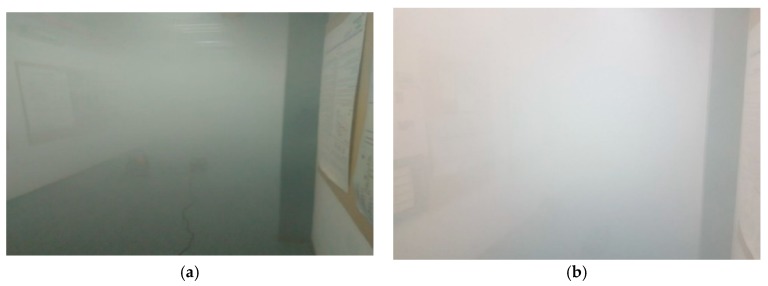
(**a**) The process of filling the corridor with fog, the visibility of about 1.5 m; (**b**) The corridor is filled with fog until the visibility was less than a meter.

The initial thermal conditions along the corridor were 16 °C and 70% relative humidity. The process of filling the space with the artificial fog lasted several minutes to reach a visibility less than one meter. The filling process can be seen in Figure 6. The droplet number concentration $n_{d}$ and liquid water content $W_{0}$ can be estimated using expression (17). For a visibility of Vis=0.5 m we found that in our case, $n_{d}=4000$ cm^−3^ and $W_{0}=30$ g/m^3^.

## 6. Verification of the Theory

**Figure 7:**
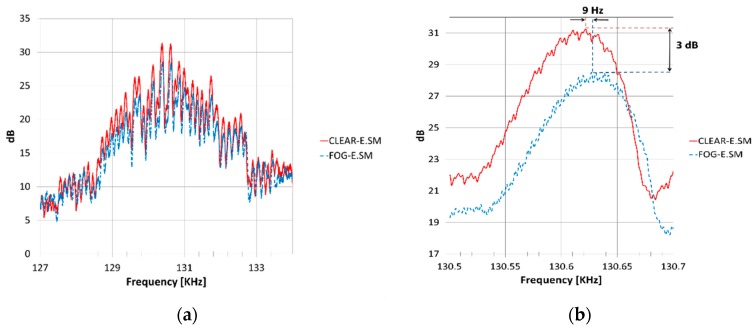
(**a**) The detected intermediate frequency (IF) signal spectrum when the target is at a physical distance of 18.8 m in both cases with and without fog; (**b**) after filtering and smoothing.

The experiments with the 320–330 GHz FMCW radar were carried out without fog, as a reference background case, and with fog where the visibility was 0.5 m. The output signal at IF was recorded in the form of power spectral density in the spectrum analyzer. The signal obtained contained relatively large spectral fluctuations, mainly due to multi reflections from different objects along the corridor, as can be seen in Figure 7a. Knowing the physical distance of the target, we perform numerical smoothing algorithm. The detected spectrum measured in a clear corridor (without fog) and that obtained in the presence of fog are both shown in Figure 7b.

When the fog is introduced, 3 dB attenuation and 9 Hz frequency up-shift are revealed in the IF signal in respect with the reference (no fog) measurement. These results correspond to an attenuation of 80 dB/km and a time delay of 230 ps/km. Using the theoretical model, we evaluated these values as summarized in Table 3.

Simulations were carried out for reference conditions as prevailed during the experiment; ambient temperature 16 °C, air pressure 101 kPa and relative humidity *RH* = 70%. Some small differences between simulation results and experimental measurements were noted. In order to demonstrate the effects pointed out theoretically, in a limited experimental space (a corridor with a length of about 20 m), we generated a heavy fog and brought the visibility to be very low. This may lead to some uncertainties in the visibility estimation, mainly due to extremely low, hard-to-measure visibility of the artificial fog along the whole corridor. However, the typical droplets dimension generated by the ‘fog machine’ were small enough to be well in the Rayleigh scattering regime even if the temperature changes during the experiment. This enables neutralization of temperature fluctuation and its effect on the diameter of the droplets on the scattering phenomena even in sub-millimeter wavelengths.

According to expression (21), a frequency shift of $\Delta f_{IF}=9\;\mathrm{Hz}$ corresponds to an incremental group delay of $\Delta \tau_{d}=4.32\;\mathrm{ps}$. This means that the fog introduces additional delay in the received signal with respect to the physical distance measures at clear conditions. The resulted time delay enables calculation of the index of refraction of the fog at 320 GHz. Using relations (15), we estimated the real part of refractivity in the presence of the fog to be $N_{0}+N^{\prime }\left(f\right)=68\;\mathrm{ppm}$. From the measured attenuation of 3 dB, we also estimated the imaginary part of the refractivity to be $N^{\prime \prime }\left(f\right)=50\;\mathrm{ppm}$.

## 7. Summary and Conclusions

The paper discussed the absorptive and dispersive characteristics of fog and their effects on the accuracy of a radar operating in the millimeter wave regime. A theoretical analysis of the attenuation and group delay emerged due to the suspension of water droplets in the atmosphere, occurring in foggy weather conditions. We derive the relations between the optical visibility in foggy conditions and the dielectric properties of the atmospheric medium, as well as its complex refractivity.

The effects of the fog on radar accuracy are demonstrated by generating fog artificially with a fog machine. The resulted heavy fog produced in a closed corridor enables an experimental realization of the theory even in a relatively short distance. The dimensions of the generated fog droplets were small enough to keep the millimeter wave scattering well within the Rayleigh regime and neutralize variations in the environmental conditions, such as temperature and pressure that may affect droplet sizes. Small droplet dimensions enabled us to distribute the fog quite uniformly along the corridor, and bring the visibility to be low enough to demonstrate absorption and dispersion effects caused by the suspended small droplets.

The measurements were done using high resolution FMCW radar operating at 330 GHz. The high bandwidth of the radar and its extended frequency sweep, allow us to reveal the effects quantitatively. The experimental measurements showed an agreement with the calculated results predicted by the theory.

## Figures and Tables

**Table 1:** Comparison of measured fog attenuation in millimeter wavelengths with previous researches.

Frequency (GHz)	Reference	$\mathrm{Water}\;\mathrm{Droplet}\;\mathrm{Concentration}\;W_{0}$ (g/m^3^)	Fog Attenuation Experiment (dB/km)	Fog Attenuation Simulation (dB/km)
72.56	[24]	0.2	0.6	0.7
210	[25]	0.03	0.4	0.3
240	[26]	3	37	38.6

**Table 2:** 320–330 GHz FMCW radar parameters.

Starting frequency	$f_{0}$	320 GHz
Sweep bandwidth	BW	10 GHz
Sweep time	$T_{s}$	9.6 ms
Transmitted power	$P_{t}$	10 dBm
Antenna gain	G	40 dBi
Beam width	$\theta_{beam}$	1.3°
Polarization	Linear	Horizontal

**Table 3:** Simulation and experimental results for frequency of 320 GHz at distance *d* = 18.8 m.

Parameter Name	Parameter Symbol	Units	Model			Experiment
			No Fog	Fog	Change	Difference
Visibility	Vis	(m)	∞	0.5		
Water content	$W_{0}$	(g/cm^3^)	0	30		
Attenuation	20log(e)⋅α(f)	(dB)	0.35	2.1	1.75	1.5
Intermediate frequency	$f_{IF}$	(Hz)	130,622	130,631	9	9
Group delay	$\tau_{d}$	(ps)	62,752	62,756	4	4.3
Incremental group delay	$\Delta \tau_{d}$	(ps)	2710	2714	4	4.3
